# Rhein: A Review of Pharmacological Activities

**DOI:** 10.1155/2015/578107

**Published:** 2015-06-22

**Authors:** Yan-Xi Zhou, Wei Xia, Wei Yue, Cheng Peng, Khalid Rahman, Hong Zhang

**Affiliations:** ^1^Central Laboratory, Shanghai Seventh People's Hospital, Shanghai 200137, China; ^2^Key Laboratory of Standardization of Chinese Herbal Medicines of Ministry of Education, Pharmacy College, Chengdu University of Traditional Chinese Medicine, Chengdu 610075, China; ^3^Department of Nuclear Medicine, Shanghai Seventh People's Hospital, Shanghai 200137, China; ^4^School of Pharmacy and Biomolecular Sciences, Faculty of Science, Liverpool John Moores University, Liverpool L3 3AF, UK; ^5^Department of Pharmaceutical Botany, School of Pharmacy, Second Military Medical University, Shanghai 200433, China

## Abstract

Rhein (4, 5-dihydroxyanthraquinone-2-carboxylic acid) is a lipophilic anthraquinone extensively found in medicinal herbs, such as *Rheum palmatum* L., *Cassia tora* L., *Polygonum multiflorum* Thunb., and *Aloe barbadensis* Miller, which have been used medicinally in China for more than 1,000 years. Its biological activities related to human health are being explored actively. Emerging evidence suggests that rhein has many pharmacological effects, including hepatoprotective, nephroprotective, anti-inflammatory, antioxidant, anticancer, and antimicrobial activities. The present review provides a comprehensive summary and analysis of the pharmacological properties of rhein, supporting the potential uses of rhein as a medicinal agent.

## 1. Introduction

**Figure 1:**
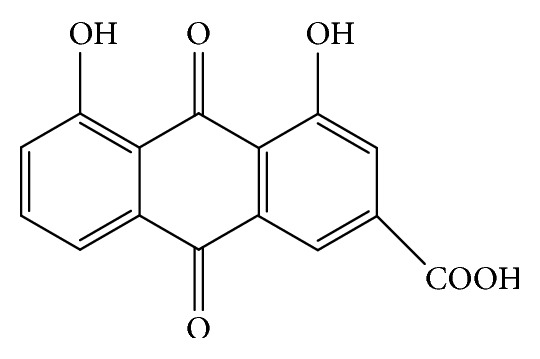
Chemical structure of rhein.

Rhein (4,5-dihydroxyanthraquinone-2-carboxylic acid, Figure 1) is a lipophilic anthraquinone extensively found in medicinal herbs* Rheum palmatum *L.,* Cassia tora* L.,* Polygonum multiflorum *Thunb. and* Aloe barbadensis* Miller, and so on, which have been used medicinally in China for more than 1,000 years. Diarrhea, the most common side effect, is well tolerated in humans. Rhein exhibits linear pharmacokinetics between 50 and 200 mg [1] and has many pharmacological effects, including hepatoprotective, nephroprotective, anti-inflammatory, antioxidant, anticancer, and antimicrobial activities (summarized in Table 1). These pharmacological effects lay the foundation for the treatment of hepatic disease [2], osteoarthritis [3], diabetes [4], atherosclerosis [5], and various cancers, such as nasopharyngeal carcinoma [6], tongue cancer [7], hepatocellular carcinoma [8], and lung cancer [9]. The aim of the present review was to give a comprehensive summary and analysis of the pharmacological properties of rhein, supporting the potential uses of rhein as a medicinal agent.

## 2. Pharmacology

### 2.1. Hepatoprotective Activity

Rhein has been shown to modulate cytochrome P450 (CYP) enzymes in rat liver microsomes. For example, rhein significantly inhibited CYP2E1; inhibition constant (*Ki*) = 10 *μ*m (mixed); CYP2C9 and CYP3A were also inhibited evidently;* Ki* = 38 *μ*m (mixed) and* Ki *= 30 *μ*m (mixed), respectively; but rhein revealed only mild inhibitory effects on CYP1A2 (*Ki* = 62 *μ*m, uncompetitive) and CYP2D6 (*Ki* = 74 *μ*m, mixed) [10].

In hepatitis B virus-transgenic mice with nonalcoholic steatohepatitis induced by a high-fat (HF) diet, rhein was found to attenuate the serum levels of total cholesterol, triglyceride, and fasting plasma glucose, ameliorating glucose and lipid metabolism [11]. Oral administration of rhein significantly accelerated energy expenditure and decreased the levels of cholesterol and liver triglyceride. It lowered body weight, the expression of the lipogenic enzyme sterol regulatory element-binding protein-1c (SREBP-1c) and its target genes in liver, and the transcriptional activity of SREBP-1c through its upstream regulator, liver X receptor (LXR). Rhein also improved insulin resistance and hepatic steatosis and normalized alanine aminotransferase (ALT) levels in HF diet-induced obese mice. Moreover, rhein regulated the T helpers Th1/Th2 responses by inhibition of T-box expressed in T-cells (T-bet) expression and enhancement of GATA-binding protein-3 expression through increased signal transducer and activator of transcription 6 phosphorylation [12].

Fibrosis, characterized by extracellular matrix accumulation and disruption of normal tissue structure, is a common cause of chronic failure of many organs [13]. The recent evidence supports rhein as an antifibrotic agent in hepatic disorders. In carbon tetrachloride/ethanol-induced liver fibrosis rats, rhein downregulated the levels of serum ALT, hyalauronic acid, procollagen type III, and liver malondialdehyde (MDA), upregulated the liver superoxide dismutase (SOD) level, and inhibited the expression of transforming growth factor beta 1 (TGF-*β*1) and alpha-smooth muscle actin (*α*-SMA), the collagen staining positive area and the grade of fibrosis in the liver [2]. Furthermore, rhein markedly improved histological changes of fibrosis and attenuated the expression of *α*-SMA and TGF-*β*1 in the liver, suggesting its protective effect from hepatocyte injury and hepatic fibrosis [14].

### 2.2. Nephroprotective Activity

Several researches have demonstrated the nephroprotective property of rhein both* in vivo* and* in vitro*. In Sprague-Dawley rats with immune globulin anephropathy (IgAN), rhein enhanced the expression of intestinal epithelial tight junction proteins zona occludens protein-1 and occludin, repaired damaged tight junctions, and protected the intestinal barrier [15]. Oral administration of rhein (150 mg/kg/d) evidently ameliorated renal interstitial fibrotic lesions and attenuated the expression of *α*-SMA and deposition of fibronectin (FN) in mice with renal interstitial fibrosis induced by unilateral ureteral obstruction. Rhein also suppressed TGF-*β*1 and its type I receptor expression in obstructed kidneys.* In vitro*, rhein abolished the *α*-SMA and FN expression in rat kidney interstitial fibroblasts cells (NRK-49F) induced by TGF-*β*1, suggesting that rhein is a potent inhibitor of renal interstitial fibrosis [16].

Rhein markedly ameliorated the glomerular hypertrophy, mesangial expansion, excessive extracellular matrix, and renal capsule dilation in IgAN rats. Additionally, rhein administration evidently decreased IgA deposition in glomerulus, the volume of urinary red blood cells, 24-h urinary protein excretion, and the expression of upregulated FN and *α*-SMA in renal tissue [17]. In chronic allograft nephropathy rat models, rhein improved renal function through reductions of renal fibrosis and interstitial inflammation and increases of bone morphogenetic protein 7 and hepatic growth factor levels. Furthermore, both FN and collagen IV were reduced in the extracellular matrix [18].

Rhein was capable of protecting against renal injury progression and ameliorating pathological changes by regulation of the activities of nuclear factor-kappa B (NF-*κ*B) and caspase-3 in the early phase of glomerulosclerosis induced by both unilateral nephrectomy and injection with adriamycin into caudal vein in rats. One of the possible molecular mechanisms by which rhein alleviated renal tissue cell apoptosis in glomerulosclerosis is that caspase-3 expression in kidney is downregulated [19]. Furthermore, rhein inhibited the hypertrophy of renal proximal tubular epithelial cells induced by high glucose (30 mM) and angiotensin II (10^−7 ^M) in rats through significantly decreasing increased cell size, ^3^H-leucine incorporation, and cellular protein content [20].

### 2.3. Chondroprotective Activity

There has been a large amount of research on the effects of rhein on osteoarthritis (OA) chondrocytes and tissue separated from human or other animals. Interleukin-1*β* (IL-1*β*) plays a fundamental role in OA pathophysiology and cartilage destruction [3]. Several cells in articular joint tissue produce IL-1*β*, such as macrophages, synovial cells, and chondrocytes. This cytokine contributes to degeneration of articular cartilage by stimulating the cells to produce proteolytic enzymes and by decreasing the anabolism of the chondrocytes [3]. Rhein (10^−5 ^M) enhanced by 46.5% of aggrecan and 50% of prostaglandin E_2_, while it reduced by 17–30% of interleukin-6 (IL-6), matrix metalloproteinase (MMP)-3, nitric oxide (NO), and macrophage inflammatory protein-1*β* in human osteoarthritic chondrocytes incubated with 10^−10 ^M IL-1*β* [21]. Rhein markedly decreased IL-1 converting enzyme protein production [3] and partially increased tissue inhibitor of metalloproteinase-1 (TIMP-1) synthesis and NO production of IL-1*β* [22, 23]. Moreover, rhein slightly decreased monocyte chemotactic protein-1 (MCP-1) production, while it increased the levels of IL-1 receptor antagonist (IL-1RA), cytokine receptors IL-6R, soluble tumor necrosis factor (sTNF) R I and R II, and some chemokines or intercellular adhesion molecule (ICAM)-1 in IL-1 (1 ng/mL)-stimulated chondrocytes from osteoarthritic patients [24]. Rhein (5–20 mg/mL) inhibited 1,25(OH)_2_D_3_-induced osteocalcin release, urokinase plasminogen activator (u-PA) production, and plasminogen activator inhibitor (PAI)-1 levels but increased the levels of insulin-like growth factor-1, prostaglandin E_2,_ and cyclooxygenase-2 in human OA primary subchondral osteoblasts [25].

IL-1 plays an important role in the OA pathogenesis. Rhein (10^−7^–10^−5 ^M) notably blocked IL-1*β* production and NO release stimulated by lipopolysaccharide (1 *μ*g/mL) in human OA cartilage and synovial tissue cultures. Rhein also reversed the inhibitory effect of lipopolysaccharide (LPS) on cartilage ^35^S uptake and increased IL-1RA content in cartilage culture media [26]. Rhein also effectively inhibited the synthesis of IL-1*β* in human OA synovium, as well as the action of this cytokine on the cartilage, by reducing the content of chondrocyte IL-1 receptors [27].

Rhein (10^−5 ^M) had a weak action on *α*4/*β*1 or *α*5/*β*1 receptors in TNF-*α* or recombinant human IL-1*α*- (rhIL-1*α*-) stimulated chondrocytes (human chondrosarcoma cell line HEM-C55) [28]. Rhein was found to downregulate the proliferation rate of both synoviocytes and chondrocytes, decrease caspase-3/7 activities, and increase the expression of p21 and/or p27, but not cyclin D1 [29].

After bovine articular chondrocytes were cultured in low oxygen tension with rhein (10^−5 ^M) for 24 h, IL-1*β* (10 ng/mL)-activated mitogen activated protein kinase (MAPK) pathway, DNA binding of NF-*κ*B, and activator protein-1 (AP-1) were inhibited significantly. NF-*κ*B and AP-1 are two key factors related to the expression of several proinflammatory genes in chondrocytes. Furthermore, rhein could prevent the procatabolic action of the cytokine by inhibition of the collagenase synthesis and increase the synthesis of matrix components, such as type II collagen and aggrecan, which might be the mechanism of its disease-modifying effect in OA [30]. Rhein (10^−4 ^M) evidently prevented increases of MMPs and aggrecanase-1, NF-*κ*B, and AP-1 DNA binding, phosphorylation of extracellular signal-regulated protein kinase (ERK), and c-Jun NH2-terminal kinase in bovine chondrocytes stimulated by IL-1*β* (10 ng/mL)* in vitro *[31]. Rhein dose-dependently inhibited IL-1*β*-induced degradation of the inhibitor *κ*B-*α* protein, translocation of the protein p65 (a member of the NF-*κ*B family) to the nucleus, and NF-*κ*B binding to a specific (gamma-(32)P)-labelled oligonucleotide probe. Rhein also inhibited inducible NO synthase mRNA and protein synthesis and NO production in a dose-dependent manner [32]. In cultured rabbit articular chondrocytes, rhein (0.1–30 *μ*M) dose-dependently suppressed the rhIL-1*α*-induced proteoglycan degradation, MMPs activity, and the expression of proMMPs-1, -3, -9, and -13, while it increased the production of TIMP-1 [33, 34]. Rhein at 20 *μ*M inhibited the activity of cathepsin B from human liver. In cultured rabbit cartilage challenged with IL-1*β*, rhein at 100 *μ*M suppressed cathepsin B activity and proteoglycan release. After treatment with oral diacerein, the prodrug of rhein, at the dose of 25 mg/day for 3 months, the progression of OA lesions and osteophyte formation were restrained in the experimental OA rabbit model [35].

### 2.4. Anti-Inflammatory Activity

Reducing the expression of endothelial cell adhesion molecules (ECAMs) is known to decrease inflammation-induced vascular complications. The transcription and expression of ECAMs, including ICAM-1, vascular cell adhesion molecule-1 (VCAM-1), and E-SELECTIN, could be reduced by the rhein treatment (10 and 20 *μ*M) in human umbilical vein endothelial cells (HUVECs). In the presence of LPS stimulation, the transcription and expression of VCAM-1 were also inhibited by treatment with rhein (10 and 20 *μ*M) [36].

Adjuvant injection elicited the inflammatory edema in rat paw, accompanied by activation of nicotinamide adenine dinucleotide phosphate (NADPH) oxidase (p22phox gp91phox), transcript factor 6 (ATF6) and p66Shc, elevation of cytokines including MMP-2, and an increase of the p-Akt/Akt ration, which were notably reversed by rhein [37]. Rhein (20 *μ*M) almost completely inhibited intersegmental blood vessels formation at both 48 and 72 h after fertilization (hpf) and completely prevented subintestinal vessel plexus formation at 72 hpf in wild type zebra fish embryos. Rhein affected multiple molecular targets related to angiogenesis, particularly* angpt2* and* tie2*, and also blocked endothelial cell migration [69].

### 2.5. Antioxidant Activity

The superabundant production of reactive oxygen species (ROS) is involved in many pathophysiological processes such as aging, atherosclerosis, cancer, neurodegenerative disorders, chronic inflammation, and degenerative rheumatic disease. Rhein was observed to inhibit the ROS production in human peripheral neutrophils activated by N-formyl-methionyl-leucyl-phenylalanine or phorbol-12-myristate-13-acetate* in vitro* [38]. Besides, many other mechanisms of the antioxidant effect of rhein have been revealed. Pretreatment with different rhein concentrations (2, 4, 8, and 16 *μ*M) significantly downregulated the mRNA expression of Bid, caspase-3, -8, and -9 and the content of MDA and lactate dehydrogenase, while it increased NO content and activities of NO synthase, SOD, and glutathione peroxidase (GSH-PX) in hydrogen peroxide- (H_2_O_2-_) insulted HUVECs, reversing H_2_O_2_-induced cell apoptosis [39]. Rhein dramatically decreased acetaminophen-induced serum glutamate-pyruvate transaminase, glutamate-oxaloacetic transaminase, creatinine and urea nitrogen levels in the liver, ROS production, NO and MDA levels, and GSH concentration in the liver and kidney of rats. Rhein also significantly ameliorated the histopathological damage of the liver and kidney [40]. Rhein eliminated the biphasicity of ubiquinone oxidoreductase- (NADH-) induced reaction and caused a substantial stimulation of NADH-induced lipid peroxidation in beef heart submitochondrial particles. Furthermore, rhein facilitated both NADH- and NADPH-induced lipid peroxidation [41].

### 2.6. Anticancer Activity

Anticarcinogenic effects of rhein on proliferation and metastasis in cells have been investigated* in vitro*. Rhein inhibited hypertrophic scar fibroblasts proliferation in a dose-dependent manner [70]. Rhein also dose-dependently inhibited 12-O-tetradecanoylphorbol-13-acetate- (TPA-) induced cell transformation and AP-1 activation, prevented the phosphorylation of c-Jun protein and c-Jun NH2-terminal kinase (JNK), did not restrain the phosphorylation of ERK and p38 kinase in mouse epidermal cell JB6 line [42]. Rhein (0.1 and 1 mg/mL) evidently suppressed cell proliferation and mitogen-activated protein (MAP) kinase activation in human colon adenocarcinoma cells (Caco-2) but significantly lessened H_2_O_2_-induced DNA damage and the elevated MDA and ROS levels induced by H_2_O_2_/Fe^2+^ at the concentrations of 0.1–10 mg/mL [43].

Cancer invasion is believed to be dependent on extracellular matrix remodeling elicited by tumor cells. Rhein inhibited invasion and migration in human nasopharyngeal carcinoma (NPC) cells through downregulation of the expression of MMP-9, vascular endothelial growth factor (VEGF), growth factor receptor bound protein 2, son of sevenless-1 and Ras, inhibition of the phosphorylation of ERK, p38 MAPK, and activation of transcription factor NF-*κ*B [6]. Rhein prevented HUVEC tube formation, proliferation, and migration stimulated by vascular endothelial growth factor (VEGF_165_) under normoxic and hypoxic conditions. Moreover, rhein inhibited the activation of phosphatidylinositol 3-kinase (PI3K), phosphorylated-AKT (p-AKT), and phosphorylated ERK, suppressing* in vitro* angiogenesis. Rhein restrained cell cycle and viability of hormone-dependent breast cancer cells (MCF-7) and hormone-independent breast cancer cells (MDA-MB-435s) under normoxic or hypoxic conditions. In addition, rhein decreased the expression of hypoxia-inducible factor (HIF)-1*α*, VEGF_165_, epidermal growth factor (EGF), the phosphorylation of NF-*κ*B inhibitor, and the activity of heat shock protein 90*α* (Hsp90*α*) under normoxic or hypoxic conditions [44].

Rhein prevented the mRNA expression of MMP-9, which plays an important role and is the most associated with tumor invasion and metastasis in various human cancers, decreased the levels of MMP-2 and urokinase u-PA, and inhibited the migration and invasion in human tongue cancer SCC-4 cells [7]. A further study demonstrated that rhein dose-dependently induced DNA damage in SCC-4 cells, followed by the inhibition of the mRNA expression of DNA repair-associated O (6)-methylguanine-DNA methyltransferase (MGMT) [45]. The mitosis was inhibited in* Allium cepa* root tips incubated with rhein in a dose-dependent manner [71].

Apoptosis, a physiological process for eliminating malignant cells including cancer cells, does not result in the damage to normal cells or surrounding tissues. Rhein-induced apoptosis has been reported in various human cancer cells. Incubation of human hepatocellular carcinoma BEL-7402 cells with rhein at 50–200 *μ*M for 48 hours caused an increasing apoptosis, the features of which included cellular morphological change and chromatin condensation. Additionally, rhein induced cell cycle S-phase arrest, decreased c-Myc gene expression, and increased caspase-3 gene expression [8]. Rhein induced the abrogation of mitochondrial membrane potential and cleavage of Bid protein in human cervical cancer Ca Ski cells. Rhein decreased the level of Bcl-2 while increased the levels of Fas, p53, p21, Bax, and cytoplasmic Ca^2+^ and the activities of both caspase-8 and -9, promoted caspase-3 activation, and resulted in DNA fragmentation [46].

Rhein induced apoptosis in human promyelocytic leukemia cells (HL-60) through facilitating the loss of mitochondrial membrane potential, cytochrome c release from mitochondrion to cytosol, and cleavage of Bid protein. Rhein also increased the generation of ROS and the phosphorylation of c-Jun N-terminal kinase and p38 kinase [47]. Rhein elevated nuclear condensation and DNA fragmentation, resulting in apoptosis of human NPC cells. Furthermore, rhein increased the activation of caspase-3, -8, -9, and -12 as well as the levels of glucose-regulated protein 78 (GRP 78), PKR-like ER kinase, ATF6, and CCAAT, induced the rapid accumulation of calcium (Ca^2+^), and lessened the mitochondrial membrane potential. Then Cytochrome c and apoptosis-inducing factor were released [48].

Incubation of human CaCo-2 monolayer cells with 50 *μ*M rhein induced nitrate production and a time-dependent polymorphonuclear leukocytes chemotaxis. Overnight rhein incubation produced an increasing number of apoptotic cells in the culture supernatant [49]. The treatment with 30 *μ*M rhein for 24 h showed the most efficient apoptosis induction in SCC-4 cells. Rhein inhibited p53, cyclin A and E, resulting in S-phase arrest of the cells. The ratio of Bax/Bcl-2 was changed by rhein through inhibition of Bcl-2 level. Rhein increased ROS production and Ca^2+^ release, decreased the mitochondrial membrane potential level, and activated caspase-3, -8, and -9 [50]. Rhein significantly increased the protein expression of p53 and p21/WAF1 and the levels of CD95 and its two forms of ligands, membrane-bound CD95 ligand and soluble CD95 ligand, in human hepatoblastomaG2 (HepG2) cells, which not only inhibited HepG2 cell growth but also induced cell apoptosis [51]. The IC_50_ values of rhein for KB, hepatoma BEL-7402, and mammary carcinoma MCF-7 cells were 11.5, 14.0, and 18.4 mg/mL, respectively. In KB cells treated with rhein for 96 h, the increase of 71% was observed in apoptotic cells [52]. The apoptosis was observed when A-549 human lung cancer cells were incubated with rhein at 50 *μ*M for 12 h and up to 72 h. Rhein induced G_0_/G_1_ arrest through inhibition of cyclin D3, Cdk4, and Cdk6. Rhein promoted ROS and Ca^2+^ production, capase-3 activation, and cytochrome c release from mitochondria, increased the levels of GADD153 and GRP78, both hallmarks of endoplasmic reticulum stress, induced the loss of mitochondrial membrane potential (ΔΨm), and led to apoptosis in A-549 human lung cancer cells. Rhein also increased the levels of p53, p21, and Bax, while it reduced the level of Bcl-2 [9].

### 2.7. Antidiabetic Activity

*In vivo* (in db/db mice), a significant decrease in area under curve (AUC) of glucose concentrations, simultaneously, and increases in AUC of insulin level and first-phase insulin secretion were observed after administration of rhein (120 mg/kg) for 8 weeks. Furthermore, rhein treatment greatly protected *β* cell mass and inhibited *β* cell apoptosis [53, 54]. Oral administration of rhein (120 mg/kg) for 8 or 16 weeks notably reduced fasting blood glucose level and improved glucose tolerance. After localized at *β*-cell mitochondria, rhein could protect mitochondrial ultrastructure from hyperglycemia-induced mitochondrial fission protein dynamin-related protein 1 expression, resulting in inhibition of *β*-cell apoptosis [55]. Intragastric treatment with rhein (120 mg/kg) significantly downregulated blood glucose concentrations at 0, 30, 60, and 120 min after glucose load, suppressed pancreatic *β*-cell apoptosis, and elevated the early-phase insulin secretion in mice, suggesting the potential of rhein as a novel therapeutic agent for type 2 diabetes [4].

Rhein decreased urinary albumin excretion, extracellular matrix level, and TGF-*β*_1_ and FN expression in renal tissue and also reduced the plasma levels of cholesterol, triglyceride, low-density lipoprotein cholesterol (LDL-C), and ApoE in db/db mice with diabetic nephropathy (DN) [72]. In rat mesangial cells transfected with human glucose transporter 1 (GLUT 1) gene, rhein dose-dependently decreased 2-deoxyglucose uptake, reversed cell hypertrophy, and lowered the enhanced glutamine: fructose-6-phosphate aminotransferase activity of the human GLUT 1 gene, suggesting an inhibitory effect on the GLUT 1 overexpression in diabetic nephropathy [56].

It was observed that rhein significantly lowered the secretion of FN and inhibited the proliferation of human mesangial cells in mimic hyperglycemic environment of diabetic nephropathy, the possible mechanism of which might be related to suppression of the bioactivities of TGF-*β*1 and p38MAPK [57]. TGF-*β*_1_ stimulates the glucose uptake by enhancing the GLUT 1 mRNA expression in both human and rat glomerular mesangial cells, which could be antagonized by rhein [58, 59].

Rhein strongly inhibited the uptake of both 2-deoxyglucose and 3-O-methylglucose in Ehrlich ascites tumor cells by alteration of membrane-associated functions [60]. In addition, rhein greatly decreased the induction of ROS in both the NIT-1 cells and isolated islets. Rhein enhanced insulin-stimulated glucose uptake in 3T3-L1 adipocytes, while it decreased triglyceride accumulation in streptozotocin-induced diabetic mice [61]. Rhein markedly attenuated the increased glucose uptake and GLUT1 mRNA expression stimulated by TGF-*β*_1_ in a dose-dependent manner in human glomerular mesangial cells [62]. Rhein reversed the abnormal changes of MMP-9/TIMP-1 ratio and impeded overexpression of integrin-linked kinase in high glucose-induced epithelial-mesenchymal transition of HK-2 cells [73].

### 2.8. Antimicrobial Activity

Like many herbal monomer, rhein has a potential antibacterial property. For example, rhein inhibited Arylamine N-acetyltransferase activity and growth in the bacterium Helicobacter pylori from peptic ulcer patients [63]. In the other* in vitro* study, rhein showed a good antibacterial activity against all 21 tested* staphylococcus aureus* (S. aureus) strains. 28 transporter genes of* S. aureus* ATCC25923 were differentially regulated by rhein. In particular, rhein increased the transcription of genes (srtB and isdABCDEFGI) encoding iron-regulated surface determinants system and genes (nrdIEF and nrdDG) involved in ribonucleotide reductase systems, while it prevented the transcription of genes (pflAB, nirBDR, narGH, Idh1, COL-SA0660, COL-SA2363, and COL-SA2386) responsible for anaerobic respiration and fermentation [64].

Bacterial DNA/CpG DNA is recognized as a key molecule during the pathogenesis of sepsis. Therefore, preventing CpG DNA from binding to its receptor is considered as the most promising strategy. Rhein was demonstrated to have high affinity for CpG DNA. It could significantly reduce CpG DNA- and LPS-induced TNF-*α* release in RAW264.7 cells [74].

### 2.9. Purgative Activity

Rhein (4 × 10^−3 ^M) reduced net H_2_O and Na^+^ absorption in rat colon* in-situ*. However, its secretory effect was associated with neither inhibition of Na^+^ and K^+^-ATPase nor damage of the colon epithelium [65]. Mucosal or serosal application of rhein (10 nM–0.5 mM) activated chloride secretion by excitation of submucosal neurons and release of acetylcholine and endogenous prostaglandins [66]. In addition, rhein induced ion secretion in human CaCo-2 monolayer cells [49].

### 2.10. Lipid-Lowering Activity

LXRs play important roles in regulating cholesterol homeostasis and lipid and energy metabolism. After bounding directly to LXRs in C57BL/6J mice fed a HF diet, rhein suppressed the expression levels of LXR target genes in both 3T3-L1 and HepG2 cells* in vitro*. In white adipose tissue, muscle, and liver, rhein reprogrammed the expression of LXR target genes related to adipogenesis and cholesterol metabolism. Rhein activated uncoupling protein 1 (UCP1) expression in brown adipose tissue (BAT) in wild-type mice, suggesting that rhein may protect against obesity and related metabolic disorders through LXR antagonism and regulation of UCP1 expression in BAT [67]. Rhein downregulated the mRNA levels of adipogenesis-specific transcription factors PPAR*γ* and C/EBP*α* and their downstream target genes involved in adipocyte differentiation, such as CD36, AP-2, and acyl CoA oxidase in both 3T3-L1 preadipocytes and C57BL/6 mice. Furthermore, the expression of C/EBP*β* was reduced by rhein in 3T3-L1 preadipocytes. HF diet-induced weight gain and adiposity were reversed by rhein in C57BL/6 mice [68].

### 2.11. Other Activities

Rhein was found to have estrogenic activity and the EC_50_ value was 18.96 *μ*g/mL in the yeast-based estrogenicity assay system [75]. LIGHT is known to act as a novel mediator for atherogenesis. Rhein inhibited LIGHT-induced human monocyte migration, ROS generation, the expression of chemokine receptor (CCR)1, CCR2, and ICAM-1, the production of IL-8, MCP-1, TNF-*α*, and IL-6, and the activation of the p38 MAPK and NF-*κ*B [76]. Vascular smooth-muscle cell proliferation plays an important role in atherosclerosis and restenosis. Treatment with rhein resulted in the induction of apoptosis, release of cytochrome c into the cytosol, loss of mitochondrial membrane potential, decrease in Bcl-2 and Bcl-xL, and the increase in Bax and Bak expression in TNF-*α*-induced human aortic smooth-muscle cells (HASMCs), thus inhibiting the proliferation of these cells [5].

Rhein had a potent (>76%) inhibitory effect on mast-cell degranulation in rats at a 5 mg/kg dose and suppressed lipoxygenase (LOX) enzyme activity with the IC_50_ value of 3.9 *μ*g/mL, proposing that rhein has antiallergic activity [77]. Rhein dose-dependently blocked the increase of PAI-1 mRNA expression and protein production and inhibited the activity of phosphor-p44/p42 MAP kinase induced by TGF-*β*_1_ in human endothelial cells, suggesting the protective effect on the endothelial dysfunction. Rhein might be a potential agent for the treatment of vascular diseases [78]. Furthermore, rhein inhibited IL-1-induced secretion of MMPs and aggrecanases and apoptosis in intervertebral disc cells, which prevented the progression of intervertebral disc degeneration [79].

## 3. Conclusion

As reviewed here, different pharmacological experiments in a number of* in vitro* and* in vivo* models have convincingly demonstrated the abilities of rhein to exhibit hepatoprotective, nephroprotective, anti-inflammatory, antioxidant, anticancer, and antimicrobial activities, lending support to the rationale behind several of its potential medicinal uses.

However, further studies need to be carried out in order to explore the concealed areas. Although various bioactivities of rhein are substantiated by using laboratory animal or cell models, the molecular mechanisms and targets involved are still unknown, which will count against further clinical applications of this agent.

## Figures and Tables

**Table 1:** Summary of the *in vitro *and* in vivo* evidence for the biological activities of rhein.

Pharmacological effect	*In vitro* evidence	*In vivo* evidence
Hepatoprotective activity [2, 7, 10–12]		It modulates cytochrome P450 enzymes, protects hepatocyte from injury and prevents the progress of hepatic fibrosis in rats, alleviates glucose and lipid metabolism, increases energy expenditure, and restrains proinflammatory cytokine expression in mice.

Nephroprotective activity [15–20]	It abolishes the *α*-smooth muscle actin (*α*-SMA) and fibronectin expression of NRK-49F cells.	It alleviates renal fibrosis in mice. It reduces intestinal permeability and protects the intestinal mucosa in immune globulin A nephropathy (IgAN), halts the progression of IgAN, prevents the development of glomerulosclerosis, improves renal function, reduces renal fibrosis and interstitial inflammation, and inhibits the hypertrophy of renal proximal tubular epithelial cells in rats.

Chondroprotective activity [3, 21, 25, 26, 28–35]	It inhibits cytokines (IL-1*β*, LPS, TNF-*α*, and rhIL-1*α*)-induced effects in human osteoarthritic (OA) chondrocytes, human chondrosarcoma cell line HEM-C55, human OA cartilage and synovial tissue cultures, human umbilical vein endothelial cells (HUVECs), and bovine and rabbit articular chondrocytes. In particular, it stimulates aggrecan production, promotes matrix formation, decreases the production of certain proinflammatory mediators (IL-1*β*, IL-6, IL-8, and prostaglandin E_2_), corrects the matrix metalloproteinases/metalloproteinases imbalance, decreases IL-1 converting enzyme protein production, inhibits proliferation of synoviocytes and chondrocytes, and suppresses cathepsin B activity and proteoglycan release.	

Anti-inflammatory activity [36, 37]	It reduces the transcription and expression of endothelial cell adhesion molecules.	It inhibits nicotinamide adenine dinucleotide phosphate oxidase (p22phox gp91phox) and cytokines (matrix metalloproteinase-2, activating transcript factor 6, and p66Shc).

Antioxidant activity [38–41]	Antioxidant properties in human peripheral neutrophils, HUVECs, and beef heart submitochondrial particles.	It prevents the drug-induced oxidative damage in rats.

Anticancer activity [6–9, 42–52]	Anticarcinogenic effects in mouse epidermal cell JB6 line, human colon adenocarcinoma cells (Caco-2), human nasopharyngeal carcinoma (NPC) cells, HUVECs, and tongue cancer SCC-4 cells induce apoptosis in human hepatocellular carcinoma BEL-7402 cells, human cervical cancer Ca Ski cells, human promyelocytic leukemia cells (HL-60), human NPC cells, human tongue cancer cell line (SCC-4), human hepatoblastomaG2 (HepG2) cells, KB cells, and A-549 human lung cancer cells.	

Antidiabetic activity [4, 53–62]	It inhibits transforming growth factor *β*_1_ and/or glucose transporter 1 overexpression in human and rat mesangial cells, inhibits glucose uptake in Ehrlich ascites tumor cells and human glomerular mesangial cells, and enhances glucose tolerance in 3T3-L1 adipocytes.	It decreases glucose concentrations, increases insulin secretion, and/or improves glucose tolerance in db/db mice.

Antimicrobial activity [63, 64]	Antimicrobial effects against bacterium Helicobacter pylori and staphylococcus aureus (*S. aureus*).	

Purgative activity [49, 65, 66]	It induces ion secretion in human CaCo-2 monolayer cells and stimulates electrogenic chloride secretion in guinea pig colon.	It increases Na^+^ and H_2_O flow in rat colon *in-situ*.

Lipid-lowering activity [67, 68]	It regulates cholesterol homeostasis and lipid and energy metabolism in 3T3-L1 and HepG2 cells.	It protects against obesity in mice.
